# Laboratory X-ray computed tomography imaging protocol allowing the operando investigation of electrode material evolution in various environments

**DOI:** 10.1016/j.isci.2023.107097

**Published:** 2023-06-14

**Authors:** Krzysztof Dzięcioł, Yasin Emre Durmus, Hermann Tempel, Hans Kungl, Alexander Bauer, Rüdiger-A. Eichel

**Affiliations:** 1 Fundamental Electrochemistry (IEK-9), Forschungszentrum Jülich GmbH, Jülich, Germany; 2 Institut für Materialien und Prozesse für elektrochemische Energiespeicher und wandler, RWTH Aachen University, 52074 Aachen, Germany

**Keywords:** Materials science, Materials chemistry, Materials characterization

## Abstract

A robust imaging protocol utilizing laboratory XCT is presented. Hybrid 2D/3D imaging at different scales with real-time monitoring allowed to assess, in operation, the evolution of zinc electrodes within three environments, namely alkaline, near-neutral, and mildly acidic. Different combinations of currents were used to demonstrate various scenarios exhibiting both dendritic and smooth deposition of active material. Directly from radiograms, the volume of the electrode and therefore its growth/dissolution rate was estimated and compared against tomographic reconstructions and theoretical values. The protocol combines simplistic cell design with multiple three-dimensional and two-dimensional acquisitions at different magnifications, providing a unique insight into electrode’s morphology evolution within various environments.

## Highlights

• Imaging protocol for investigation of electrode material evolution was developed
• Radiographic data allowed to capture the dynamics of the process
• Three environments were studied, namely alkaline, near-neutral and mildly acidic
• Zinc plating and/or stripping rate could be estimated using only radiograms

## Graphical abstract

**Figure:**
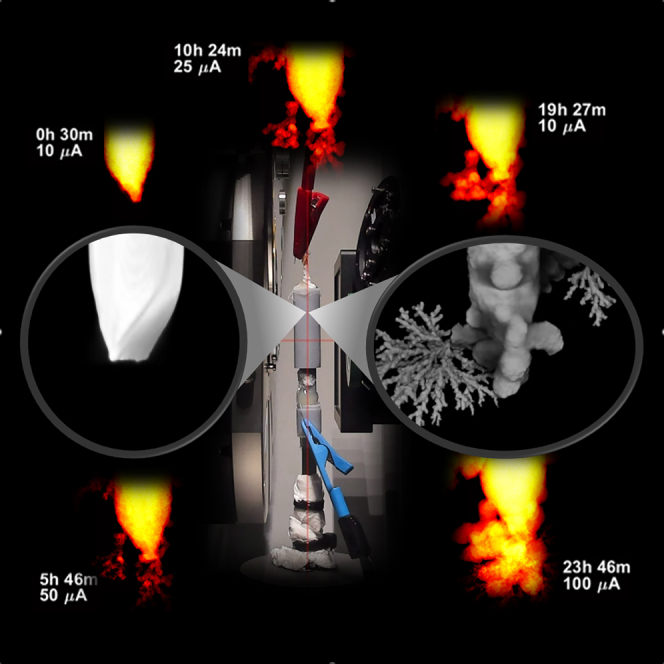

## Introduction

With the growing interest in alternative energy storage solutions, the spectrum of potential candidates to compete with lithium-ion-based systems broadens every year.^1^^,^^2^^,^^3^^,^^4^^,^^5^ This generates the need for comprehensive material testing procedures including electrochemical investigation, spectroscopy, imaging, and their combinations in the form of multimodal studies. The evolution of electrode morphology is of interest, in particular for systems that involve material dissolution and deposition, aka stripping and plating, since it reflects directly the failure mechanisms determining the performance of the complete cell.^6^^,^^7^ These mechanisms play a decisive role for rechargeable aqueous zinc-based batteries that have received significant interest due to their promising high energy densities and cost-effective, safe materials.^8^^,^^9^^,^^10^^,^^11^ Among aqueous zinc-based systems, primary batteries employing conventional aqueous alkaline electrolytes (such as Zn-MnO2, Zn-air) are so far the most advanced as they are already commercially available for various applications. Secondary aqueous zinc-based batteries (excluding Ni-Zn) are, however, still under the development stage due to facing several technical challenges. Concerning the Zn-anode, for example, shape change and dendrite growth are a few major obstacles that require deeper investigation and understanding to allow further development.^12^

The problem of 3D visualization and quantitative analysis of electrode evolution is already well exploited, however so far, only synchrotron X-ray computed tomography (SXCT) was able to capture the process dynamics.^13^^,^^14^^,^^15^^,^^16^ Possibly, the most spectacular attempt was revealed in 2015 by Finegan et al.,^17^ who was recording radiograms with a stunning rate of 1250 frames per second to capture the initiation of thermal runaway in lithium-ion battery cell. Although 1250 Hz was achieved only for radiograms, the frequency of 2.5 Hz reported for 3D tomograms was still impressive. Such ultra-fast imaging was possible at the ID15 beamline at ESRF, which focuses on maximizing the temporal resolution,^13^^,^^18^^,^^19^^,^^20^ thereby allowing *operando* testing in various scenarios.^21^^,^^22^ Similar, however, at lower frequency, hybrid 2D/3D imaging was performed by Yufit et al.^23^ There, SXCT scans were accompanied by FIB/SEM to assess the dendritic growth of metal electrode in zinc-air battery.

When aiming at high spatial resolution, synchrotron sites shine as well. For example, Tariq et al.,^24^ managed to investigate the mesocarbon microbead anodes at a voxel size of 16 nm, obviously sacrificing temporal resolution. In fact, the optimum measurement parameters are defined by the size of the sample and its environment, material attenuation coefficient, beam flux, detector resolution, and so forth. Therefore, even at a synchrotron source, it is necessary to find the compromise allowing to capture the phenomena at sufficient temporal and spatial resolution. A good example here is the study of Lewis et al.,^25^ who managed to record the interphase evolution of Li/LSPS/Li stack with a voxel size of 1.7 μm, while spending only 7 min to acquire a single tomogram.

There are many publications reporting on the electrode morphology of different materials examined by laboratory X-ray computed tomography (XCT). Unfortunately, most of the *operando* investigations had to significantly reduce the acquisition frequency^26^^,^^27^^,^^28^ or spatial resolution^29^^,^^30^ due to insufficient flux.

Using laboratory XCT, Shapovalov et al.,^27^ managed to visualize *in operando*, the crack formation in sodium iron titanate cathode, which is remarkable, especially that nano-XCT system was used, which is characterized by very limited room for the sample. Single CT however took around 20 h and was done with an experiment on hold, at fully charged or discharged states. While for the study of crack formation, it seems like a reasonable compromise, often process dynamics can’t be completely ignored. In such a case, lower spatial resolution, or degraded signal-to-noise ratio (SNR) must be accepted. Tariq et al.,^29^ managed to find the correlation between cell resistance and delamination of Si-based anode in lithium-ion battery, from tomograms with voxels as large as 9.3 μm. Most likely, actual spatial resolution was even worse since only 602 projections were used. Nevertheless, since the voids showed sufficient contrast with respect to the silicon anode and copper current collector, it was possible to assess the morphology.

When the evolution of microstructure is slow enough, even laboratory XCT provides sufficient time sampling frequency at a reasonable resolution. Probably most interesting example here, is the work of Santini et al.,^31^ who managed to visualize, in 3D, the activity of bacteria acting as a catalyst in microbial fuel-cell. The evolution of biofilm, formed by bacteria was presented within the timescale of 60 days.

It should be mentioned, following the Occam’s razor principle, that it is not always necessary to perform the measurement *in operando*. Knowledge of initial material distribution might provide enough information to predict the performance of the system, allowing for the optimization of its morphology in the future.^32^^,^^33^ Similarly, it is common to draw conclusion from *postmortem* samples, when time restraints don’t allow for *in operando* testing.^34^^,^^35^

In this work, a method for the 3D/2D quantification of active material plating and stripping by laboratory XCT is presented. This method uses four acquisition modes i.e., high-resolution-3D, low-resolution-3D, fast-high-resolution-2D, and fast-high-field-of-view-2D, providing the overview of the process as well as detailed insight into the evolution of metal electrode under different cycling conditions. Electrochemical cells with Zn electrodes were selected to demonstrate the proof-of-concept since zinc is still underrepresented in the battery industry while being a promising anodic material. The chemical composition of the electrolyte was picked as a variable, in addition to cycling parameters, allowing to present the flexibility of the method when facing different types of morphology evolution.

To the authors’ knowledge, such a flexible, multiscale, operando measurement protocol utilizing strictly laboratory device, is demonstrated for the first time.

### Method details

#### Cell design

**Figure 1:**
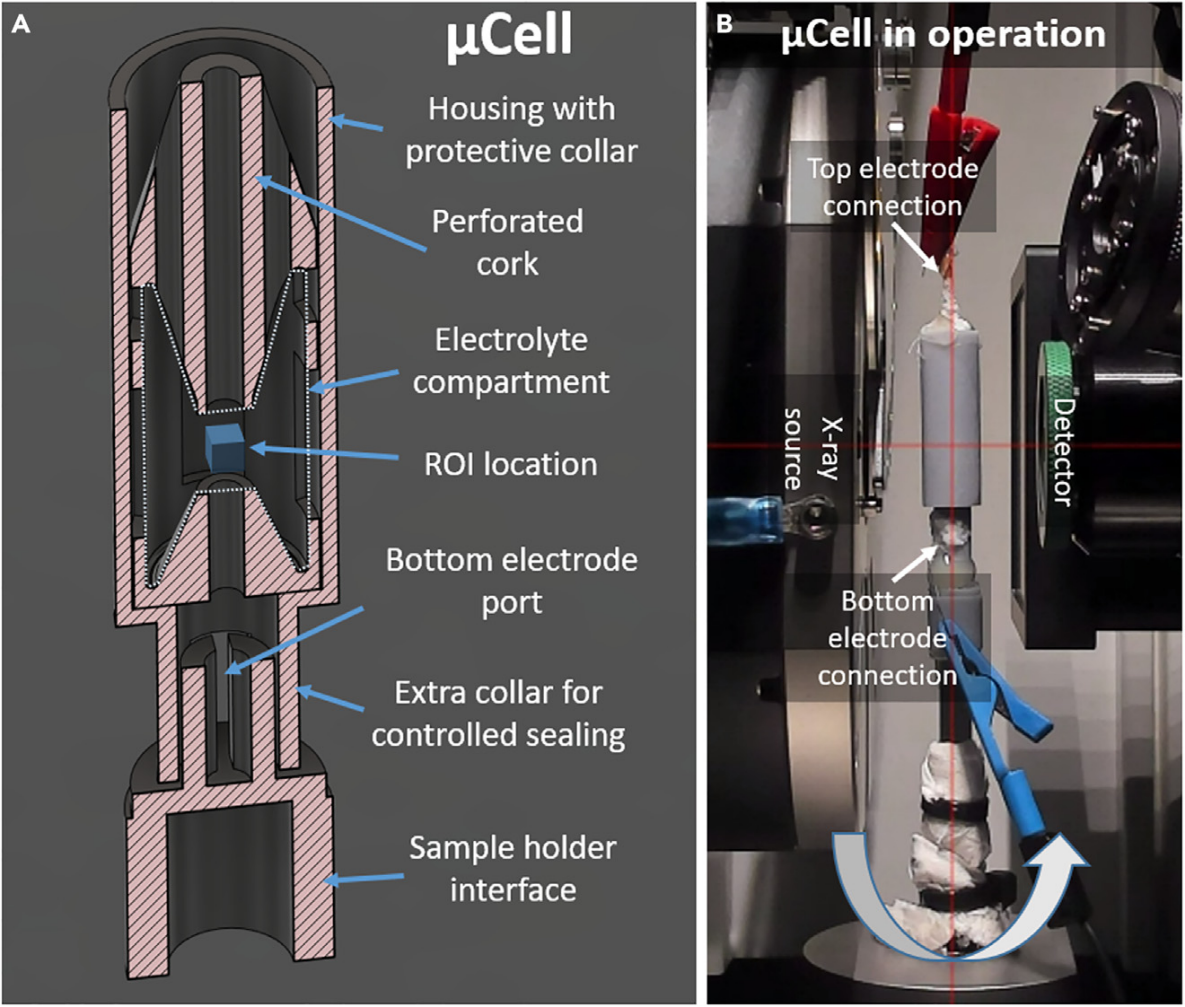
Designed cell Sectioned view of designed cell (A) and complete assembly in operation (B).

The cell was designed and 3D printed *in house* using photopolymer resin. Its sectional view is shown in Figure 1. The choice of photopolymer resin might be considered suboptimal since other materials, such as polytetrafluoroethylene (PTFE), polyetheretherketone (PEEK) or polymethylmethacrylate (PMMA), are normally preferred.^36^ Note however, that this solution is cost-effective and introduces negligible attenuation in most scenarios. Moreover, when using lower X-ray energy or imaging low-density materials (e.g. lithium), exactly the same model can be sliced for PEEK printing or even used for CNC machining. Finally, the cell is designed in a way, that allows for the outer frame to be easily replaced, e.g. by Kapton tubing. Nevertheless, at conditions applied to scan the electrodes presented in this work, attenuation caused by the thin layer of resin was negligible.

The location of the region of interest (ROI) was chosen in a way that the excess of the liquid was located above it, therefore delaying the cell failure due to electrolyte evaporation. Note that perforated “cork”, together with sloped inner walls allows gas bubbles to escape easily while preventing from building up the pressure inside the compartment. An additional collar on the top prevents the eventual spill of electrolyte during cell operation or assembly process, protecting the operator and imaging objective which can be moved very close to the cell.

#### Sample preparation

One of the aspects of XCT is its robustness. There are no special requirements for samples regarding, for instance, conductivity, magnetic permeability, surface roughness, and so forth. Moreover, samples do not need machining, although there are preferred form factors and size limitations. Therefore, it was decided to use commercially available, 0.25 mm zinc wires (Alfa Aesar, 99.99% metal basis) with circular cross-section as electrodes, without any additional machining, except cutting to the desired length. Electrodes were covered by the PEEK capillary in such a way that every time approximately the same surface area was exposed to the electrolyte. Therefore, the initial current density could be controlled. After assembling the cell, the wires were immediately immersed in the electrolyte. The aqueous electrolyte solutions were prepared by dissolving either NaCl crystals (>99.5%, Merck-Millipore), KOH pellets (>86.7%, Fluka), or ZnSO_4_ crystals in deionized water (<0.1 μS cm^−1^). NaCl-based solutions consisted of 0.5M Glycine (>99%, Alfa Aesar) and 0.2M dissolved Zn at pH 10. KOH-based solutions contained 0.2M dissolved ZnO (99.9%, Sigma-Aldrich).

#### Electrochemical testing

The two-electrode electrochemical setup consisted of two Zn wires facing each other with a distance of approximately 2 mm. The Zn wire tips, approximately 1.5 mm long, were exposed to the electrolyte. Electrochemical stripping and/or plating experiments were performed by using four different current values, i.e. - 10, 25, 50, and 100 μA, depending on the electrolyte. The detailed parameters can be found in Table 1. BioLogic SP300 potentiostat was used to control the electrochemical experiments, together with a custom Python script reading the values from potentiostat and providing an easy way to trigger 3D scans, every time when the system was switching to open circuit voltage (OCV).

**Table 1:** Sample details and protocol parameters

Label	Electrolyte	Molarity	Additives	Applied current (s) μA	Nr of 2D scans	Scan interval minutes∗∗	Nr of 3D scans	Total time h∗	Voxel size μm
zw_01	NaCl	2	Zn^2+^, Glycine	10, 25, 50, 100	214	7	2	24	0.68
zw_07	NaCl	2	Zn^2+^, Glycine	50	81	12	2	18	0.59
zw_09	KOH	6	ZnO	−100, 100	96	7	8	24	0.61
zw_10	ZnSO_4_	2	None	−100, 100, −50, 50	110	7	10	28	0.82

∗Note that total time does not include high resolution, 3D scans of pristine and postmortem samples. ∗∗Scan intervals include 5 min of waiting time and 2 min acquisition of 4 radiograms (2 at the bottom and 2 at the top electrode).

The total time of the experiment, as well as cycling parameters, were not restricted, with one exception—each combination of electrolyte, additive, and current had to have at least one cycle starting from the initial state of the electrode, i.e., having a surface without material being deposited or consumed. This way, some point of reference could be established, eliminating one variable, and allowing quick, qualitative assessment across different electrolytes and current densities. Note, however, that in a highly corrosive environment, the state of the surface might already be corrupted prior to the execution of the initial scan.

#### X-ray computed tomography imaging

Four samples, as listed in Table 1, were measured to demonstrate the robustness of the protocol. For the first sample, aka *zw_01*, the galvanostatic polarization was set to deposition mode at various currents. Sample *zw_07* was selected to demonstrate the deposition at a constant current. In both cases, the near-neutral NaCl-based (pH 10) electrolyte with glycine (0.5M) and dissolved Zn (0.2M) additives was used. *Zw_09* was tested in a conventional alkaline electrolyte (6M KOH) with ZnO additive (0.2M) under cycling conditions. Finally, *zw_10* was cycled in a mild acidic solution (2M ZnSO_4_) under two different currents.

ZEISS XRADIA Versa 620 was used to record the changes in the morphology of electrodes in both 3D and 2D. The high energy, i.e. 100 kV of X-ray tube’s voltage, together with a high-pass filter, allowed penetration through the liquid and resin with minimal interactions, while providing enough photon counts for metallic material. The loss of photons, due to the high-pass filter, was compensated by a reduction in beam hardening artifacts. The voxel size, defined by the combination of geometrical and optical (20x objective) magnification was between ∼0.6-0.8 μm (see Table 1 for exact values). The lowest resolution is reported for sample *zw_10*—due to the higher absorption of ZnSO_4_ (compared to KOH and NaCl), detector had to be moved closer to the sample to keep approximately the same acquisition time without loss of SNR.

The system in operation was measured every 5 to 10 min, only via radiograms (2D) with limited exposure (5 s) to get sufficient temporal resolution and reduce even more the eventual interactions with X-rays. Sampling interval and exposure time are critical parameters, often excluding laboratory XCT as a technique of choice. In the presented case, it was possible to predict the theoretical growth rate of the material overall (see following sections), but not on the local scale. Therefore, for setting optimal exposure, the operator has two options. First—perform a complete dummy experiment at different exposure times or second—simply choose the shortest exposure providing sufficient SNR, based on a single, 2D dummy scan. In this work, the latter was used and minimum, satisfying exposure time was 5 s with an estimated SNR∼20 dB.

3D tomograms, with 1600 projections and 10 s exposure were done *in situ* for pristine and *postmortem* states. Optionally, “low-quality,” 3D scans were triggered whenever cell operation was switched to OCV. These scans consisted of 400 projections spanning half rotation and therefore requiring a low cone angle. Moreover, OCV periods had to be long enough to cover the whole acquisition time, i.e. about 1 h. Therefore, the 3D acquisition was meant for stable systems.

**Figure 2:**
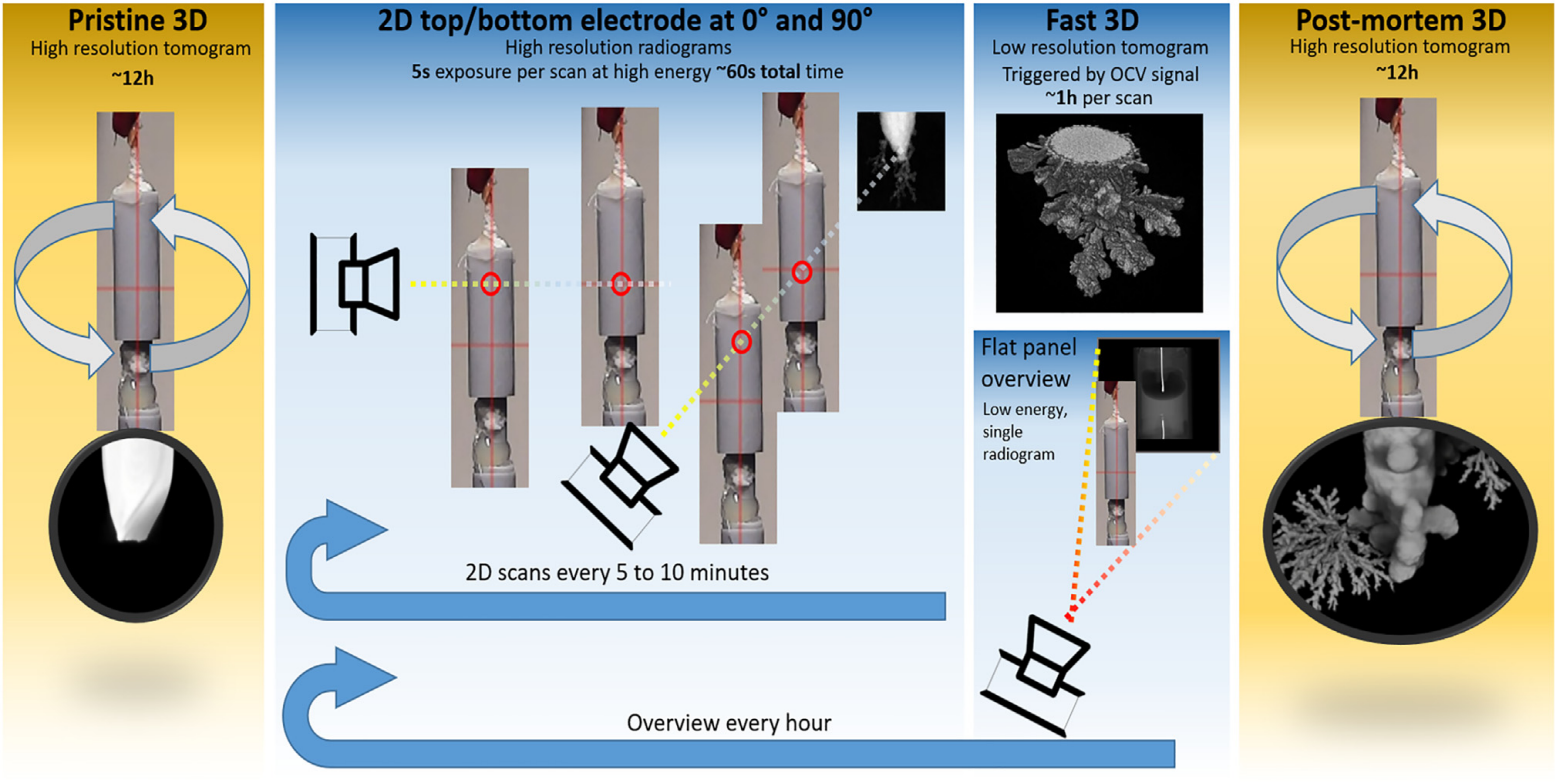
Complete protocol for material deposition studies with laboratory XCT Snapshots from pristine and postmortem tomograms belong to sample zw_01(NaCl), while low-resolution tomogram comes from the measurement of sample zw_09(KOH).

The entire measurement protocol is summarized in Table 1 and visualized in Figure 2. Note that, for better insight into the process, radiograms were recorded in two orthogonal orientations and at two different locations,—at the tip of the top and bottom wire. Additionally, one overview scan was taken after each hour of the experiment using flat-panel detector, covering the whole cell at lower energy (X-ray source voltage 40 kV) to get a good contrast between the housing, electrolyte, and air. This allowed for the inspection of bubble formation, electrolyte level, and the mechanical integrity of the cell. Furthermore, the amount of material exposed to electrochemical reaction could be estimated, which was crucial for predicting the theoretical deposition rate.

Each sample was scanned until the electrolyte level reached the exposed part of the wire or the impedance of the system raised, tripping the potentiostat’s voltage limiter (set to 1 V). Therefore, different operation times were recorded—from 18 to 28 h, depending on the applied current.

#### Custom, operando protocol development

Thanks to the exposed application programming interface (API) of the ZEISS scanner, it was possible to communicate with the device using Python and develop custom scripts for acquisition. It allowed for the automatic movement of the sample to different locations, changing of acquisition parameters such as exposure and voltage, as well as switching of lenses and filters for fully automated acquisition of 2D scans. For clarity, the protocol is presented in the form of the pseudo-code in Algorithm 1. Note that additional 3D scans were triggered manually before and after the *operando* protocol. Also, the positions at which radiograms should be acquired had to be entered manually with the help of the Scout and Scan system. This was done normally during the standard alignment of the cell, prior to custom protocol execution. More information about setting the optimal acquisition parameters can be found in the previous section (*XCT imaging*).

Algorithm 1 **Protocol implemented with XRADIA API**

Repeat:

 
Set source (100kV,14W)

 
Acquire FLAT field

 
Acquire DARK field

 
For each location/orientation:

 
Move stage (to location/orientation)

 
Scan and apply FLAT and DARK

 
If 60 minutes passed:

 
Set source (40kV, 6.5W)

 
Acquire FLAT field

 
Acquire DARK field

 
Scan and apply FLAT and DARK

 
Read current from potentiostat

 
If applied current is 0:

 
Execute 3D scan recipe

 
Reset 3D scan timer

 
Set source (off)

 
Sleep (5 minutes)

#### Real time visualization

The rapid deposition of material at higher currents might cause several problems, such as image degradation due to insufficient temporal resolution, material plating outside of the field of view, sudden bubbles agglomeration, and even mechanical degradation of the cell. Therefore, the process was visualized in real time using a custom Python script. Visualization included real time scans captured at all spatial locations, as well as overview scans. These were not used for quantitative evaluation but rather served as boundary conditions, limiting the time frame to valid acquisitions. For example, when, after a given time, electrolyte penetrates the capillary, this will inevitably lead to the creation of a new nucleation site far from the ROI. In consequence, if this event was not detected by a large field-of-view scan, the interpretation of the calculated plating/stripping rate would be strongly biased.

**Figure 3:**
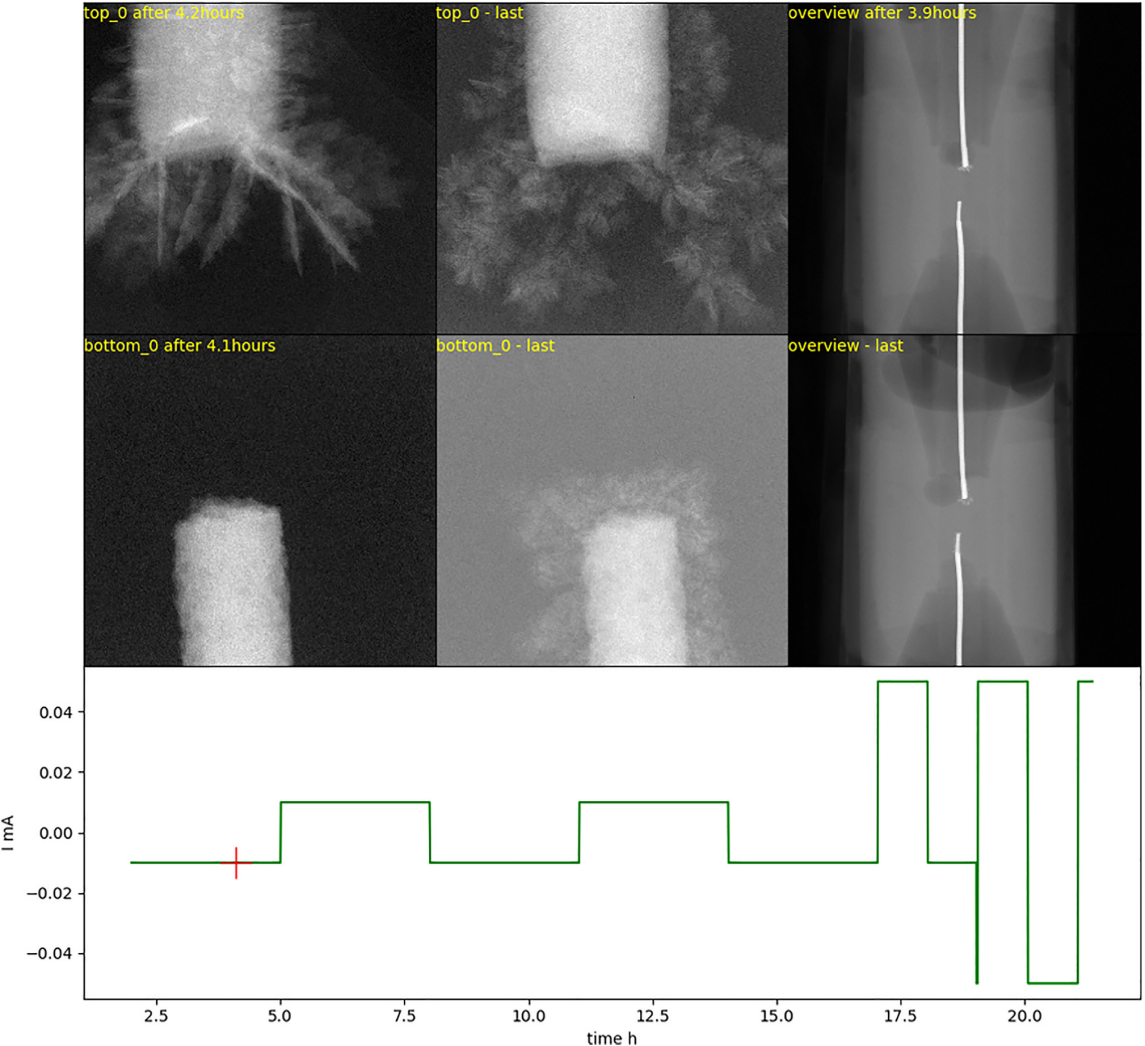
Screenshot of in house developed real time monitoring system Note that, thanks to the overview image at low energy via the flat panel, moment of bubbles agglomeration at the electrode could be captured. Moreover, the integrity of the cell and the state of the wire (appears white on the image) can be inspected.

For easier interpretation of the sample behavior with relation to the applied conditions, the imaging data was aligned with electrochemical information from the potentiostat, as it is presented in Figure 3.

#### Thickness and growth rate estimation from radiograms

Radiograms were normalized by flat and dark fields already during the acquisition. Following the Beer-Lamberts law (see Equation 1),^37^ thickness of the wire could be estimated at every pixel and time point. Note, however, that the assumption of linear dependence between thickness (x) and logarithm of intensity (I) is not valid for polychromatic radiation. There, attenuation (μ) depends not only on the Z number but also on energy (E), leading to beam hardening.

(Equation 1) $$I=I_{0}e^{-x*\mu \left(E,Z\right)}$$

A common approach to reduce the non-linear effects is acquisition at very low energy or application of pre-hardening filter (as in this work). Moreover, since we know the initial thickness of the material being penetrated (d), we can relate it to normalized intensity ($I_{n}=I/I_{0}$) as follows^37^:

(Equation 2) $$I_{n}=d\;\int_{E_{1}}\mu \left(E_{1}\right)$$

Attaching the additional block of material (Δd), requires the incorporation of the modified spectra (E_2_):

(Equation 3) $$I_{n}^{\prime }=d\int_{E_{1}}\mu \left(E_{1}\right)+\Delta d\int_{E_{2}}\mu \left(E_{2}\right)$$

Assuming that for sufficiently small Δd, energy spectra E_1_ and E_2_ are the same, we can subtract normalized intensities I_n_ and I_n_’, ignore non-linear effects and estimate the change of electrode thickness between two time points as:

(Equation 4) $$\Delta d=\frac{d}{I_{n}}\left(I_{n}^{\prime }-I_{n}\right)$$

Note that, the proportionality factor (d/I_n_) is the equivalent of calibration curves commonly used for thickness estimation in various applications^38^^,^^39^ where absolute thickness is of interest.

Finally, the growth rate can be calculated as follows:

(Equation 5) $$gr=\rho \frac{a^{2}\Delta d}{t}$$

Where *a* is the size of the isotropic voxel and ρ is the density. It can be compared directly to the theoretical growth rate (*gr*_*th*_), obtained from Faraday’s constant (*F*) for given current (*i*) and material properties (number of involved electrons *n* and molecular mass *M*):

(Equation 6) $$gr_{th}=\frac{iM}{nF}$$

Alternatively, in a more practical way, the theoretical growth rate can be derived directly from specific capacity (*C*_*s*_):

(Equation 7) $$gr_{th}=\frac{i}{C_{s}}$$

The presented approach assumes “small” changes; hence it is operating on short, linear part of such calibration curve. On the other hand, it requires good alignment of consecutive radiograms, which is critical, especially for long experiments, where significant sample drift can be observed.

#### Cross-validation using 3D information

In many cases, it is beneficial to acquire tomograms not only for pristine and *postmortem* states, but also incorporate them within the cycling routine. It allows for a better understanding of morphology evolution, especially when the surface state is significantly affected. Moreover, 3D images can be used for the cross-validation of results obtained from radiographic data. For this purpose, the estimation of the volume necessary for growth rate calculation is as simple as counting voxels in the segmented image. Precision depends on voxel size and SNR. The latter determines threshold selection, which, if picked wrongly, may result in over- or under-segmentation. Note, however, that systematic errors should cancel each other when subtracting the volumes for growth rate calculation.

Additionally, the active surface area can be estimated. In this work, standard MATLAB’s routine (The MathWorks, Inc., Natick, US), utilizing the Crofton formula was applied.^40^

#### Image processing and registration

Before the alignment, some of the radiograms had to be discarded due to the low electrolyte level at the end of the experiment or intense bubbles formation. The latter especially caused multiple frames to be removed at the beginning of each experiment since the gas trapped inside the cell during the assembly needed additional time to be released.

Registration of radiograms was performed by phase correlation (PC), which is the best choice for 2D images since it does not employ any optimization and provides a direct solution to the problem.^41^ It is, however, an intensity-based method, so it should be noted that, normally, histograms of images should be equalized prior to alignment. Although in this case, luminance variations were not expected, some basic pre-processing was done anyway. This included shifting the histograms to the first peak (background), cropping to the area around the feature of interest (wire), and applying a low-pass filter, so that the actual information was registered, not the noise.

## Results

**Figure 4:**
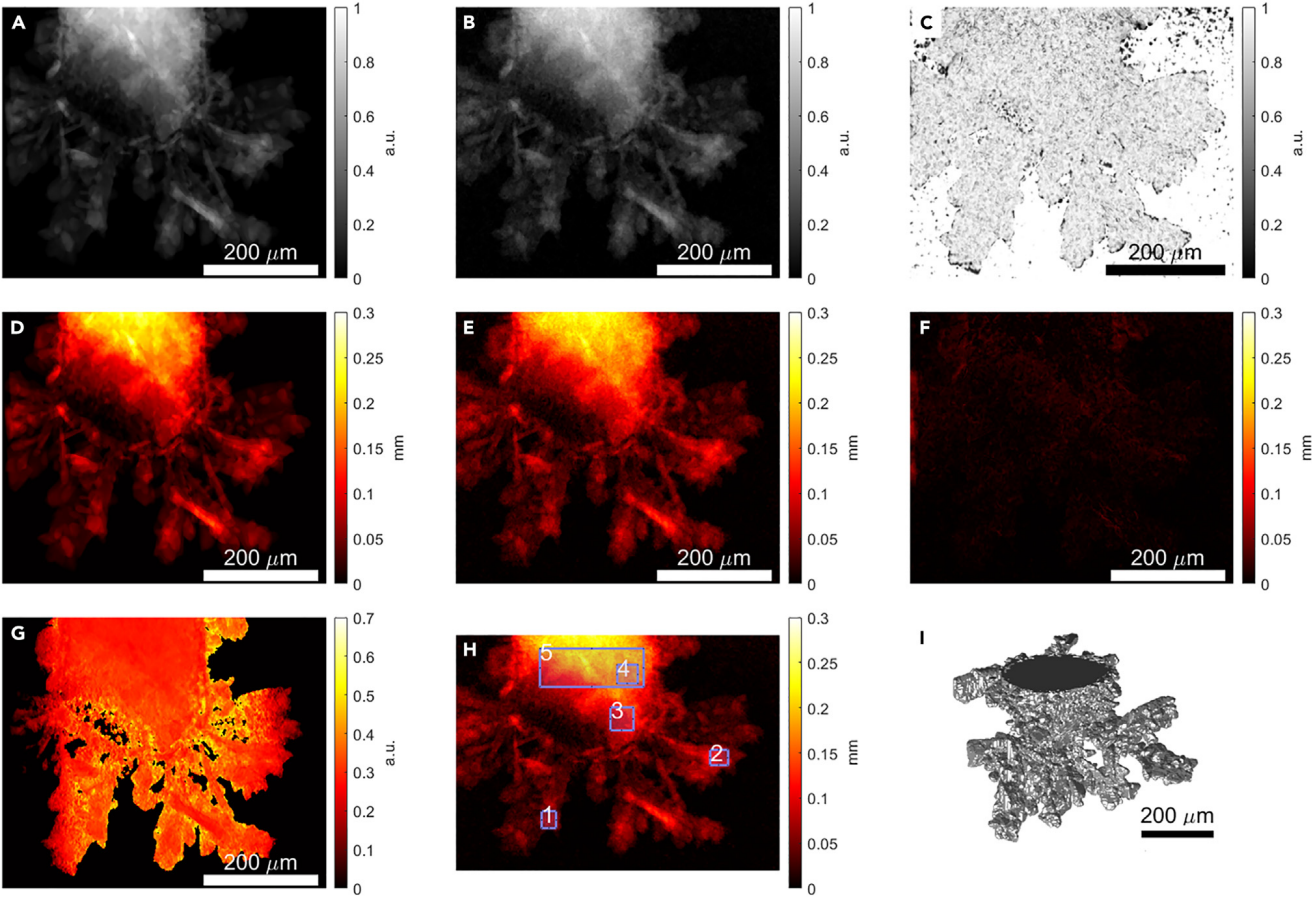
Comparison between radiographic and tomographic data Electrode image acquired as radiogram (A) and from forward projected, segmented 3D reconstruction (B) shows good overlap after PC registration as indicated by local SSI map (C)Thickness maps calculated from radiogram (D) and obtained from 3D data (E) are matching as well (F). Note that calculated proportionality factor **d/I**_**n**_ exhibits little noise at the edges (G), therefore 5 regions were selected (I) to assess local differences. 3D render (H) is presented to show that the dendrites are indeed three-dimensional.

Thanks to tomographic, three-dimensional data, thickness values calculated from radiograms, could be checked against “ground truth,” by simply comparing forward projections of acquired 3D scans to closest (in time) thickness maps. Results are presented in Figure 4. Captured radiogram, as in Figure 4A, was first aligned with corresponding, forward-projected 3D reconstruction (Figure 4B). To quantify the alignment, Structural Similarity Index was calculated (SSI = 0.87, see Wang et al.^42^) and its local distribution visualized in Figure 4C. It confirms that during OCV, when the 3D acquisition was performed, tip of the wire did not change significantly. Two thickness maps were calculated: one from the radiogram (Figure 4D), as described in the previous section, and the second directly from the 3D tomogram (Figure 4E). The latter was obtained by integrating the binarized tomogram along the dimension corresponding to 0-degree rotation angle at which the radiogram was taken. The difference image, presented in Figure 4F, shows good agreement between both maps. Proportionality factor distribution (d/I_n_), as presented in Figure 4G, which should be constant in an ideal case, exhibits elevated values only in the regions with very low attenuation, such as edges. This effect was quantified in Table 2, where estimated local thickness for several locations (see Figure 4H) was compared to results from the three-dimensional acquisition. For very small regions, the difference was as high as 16%. This does not necessarily mean that values estimated from radiograms are wrong. One can argue that the attenuation integrated through the sample should provide a more accurate thickness than the delineation of the 3D reconstruction (Figure 4I), as the latter tends to fail at the object boundaries. On the other hand, small regions will inevitably suffer from random noise generated by inelastic scattering from the liquid^43^^,^^44^—what should be more pronounced for radiograms and smoothed out in case of 3D images. This effect might be even stronger when attenuation paths elongate due to growing structures since effective SNR will drop. Nevertheless, for regions with several hundred pixels, the difference is as small as a few percent.

**Table 2:** Local volume estimation from radiograms and calculated from 3D, segmented volume

Location	3D volume	2D estimation	Relative difference
	10^4^mm^3^	10^4^mm^3^	%
1	20	20.6	3.4
2	1.98	2	4.1
3	1.44	1.7	16.3
4	0.7	0.78	10.5
5	0.4	0.48	8.7

Note that the location id corresponds to the regions highlighted in Figure 4H.

**Figure 5:**
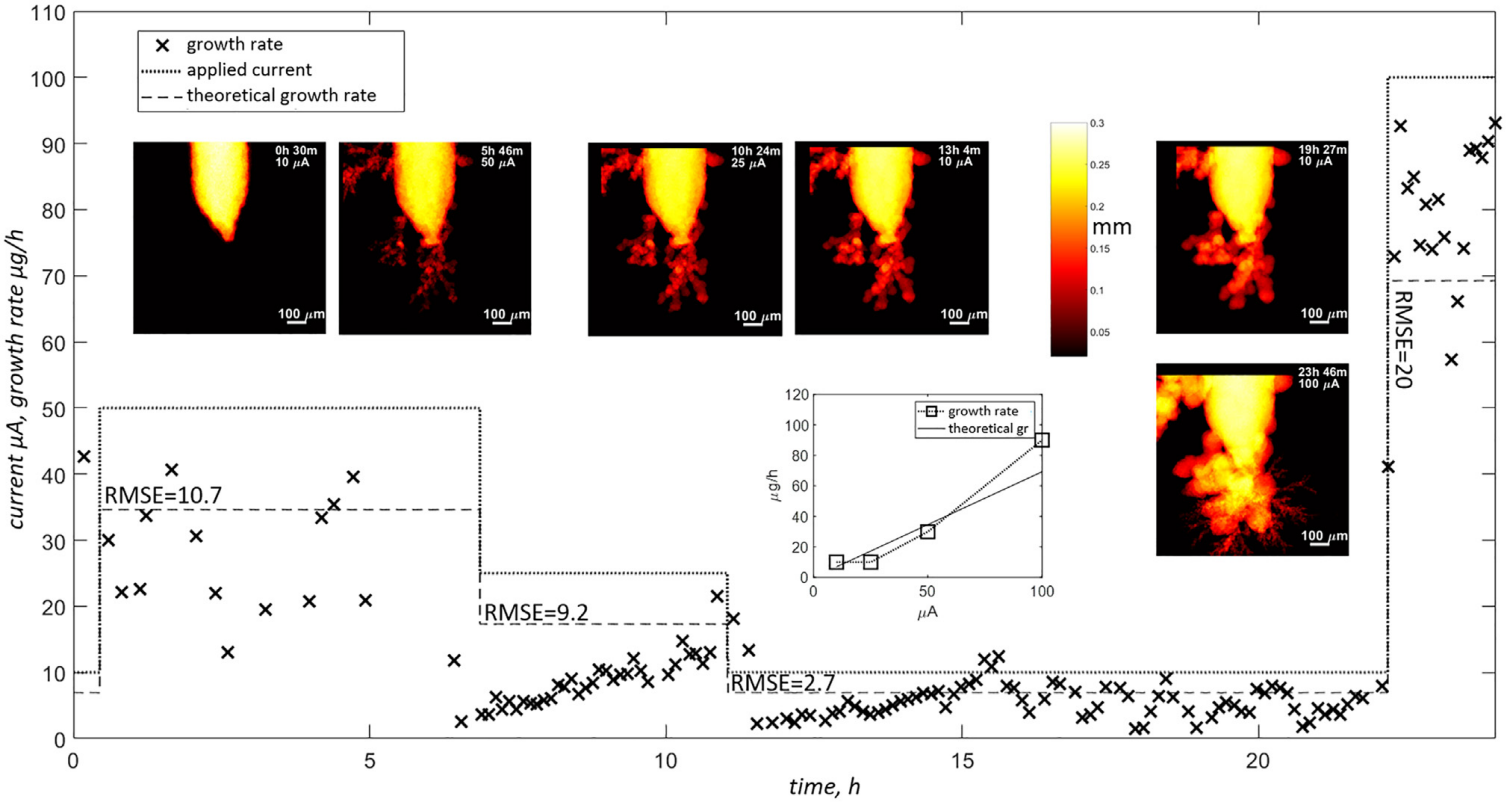
Evolution of morphology of sample zw_01(NaCl) and material growth rate vs. applied current Note the sparse sampling at the beginning of the deposition rate function (at 50μA) – operator was restarting the measurement several times and hence less points were available for calculations.

Figure 5 visualizes the deposition of zinc on *zw_01* (NaCl) electrode at different current densities over 24h. The radiograms clearly illustrate the evolution of the tip morphology at a given time and current. Initially, the deposition of Zn at 10 μA over 30 min appears to be very smooth and limited, while increasing the current to 50 μA results in considerable dendritic growth. Further deposition at reduced currents (25 and 10 μA) does not yield new nucleation sites for dendrites, but material deposition takes place at the tip and between the already grown dendrites. Although the new deposits may appear smooth, they resemble the mossy dendritic growth. Finally, the application of a relatively high current (100 μA) for a short time reveals very rapid and dendritic growth. The whole process, along with high-resolution tomograms at pristine and *postmortem* states is illustrated in Video S1

Video S1. Movie demonstrating the material plating process on zinc electrode of sample *zw_01*(NaCl)

Calculated growth rates follow the predicted material deposition rate obtained from a specific capacity of zinc (820 mAh/g) using Equation 7. Note that, on the graph, the theoretical deposition rate (gr_th_) was normalized to the length of the wire visible (L_visible_) on radiograms as in Equation 8.

(Equation 8) $$gr_{norm}=gr_{th}*\frac{L_{visible}}{L_{exposed}}$$

The assumption of linear distribution of material, especially in dendritic type of growth, may seem too simplistic at first sight. However, as long as the field of view is kept much larger than the size of individual features (dendrites), the nucleation sites, even if they appear in random locations, should span the visible part of the wire uniformly and hence promote the homogeneous deposition within the sliding window of sufficient width. In other words, the central-theorem must be applicable even if we don’t know the actual probability distribution of a single random variable, i.e., in this case, the growth rate of a single dendrite. Figure 5 confirms this by showing that after each application of new current, the measured growth rate increases until it is close to the predicted value and then oscillates around it. The periodic nature of this mechanism is exhibited even at constant currents, what is pronounced especially for lower values i.e., 25 and 10 μA. Unfortunately, it makes direct comparison to theoretical values very difficult. The root mean squared error (RMSE) varies strongly depending on the applied current and the time the system had to stabilize. For low currents and long cycle lengths, the RMSE is as low as 2.7, but it jumps up by nearly one order of magnitude for the highest current.

**Figure 6:**
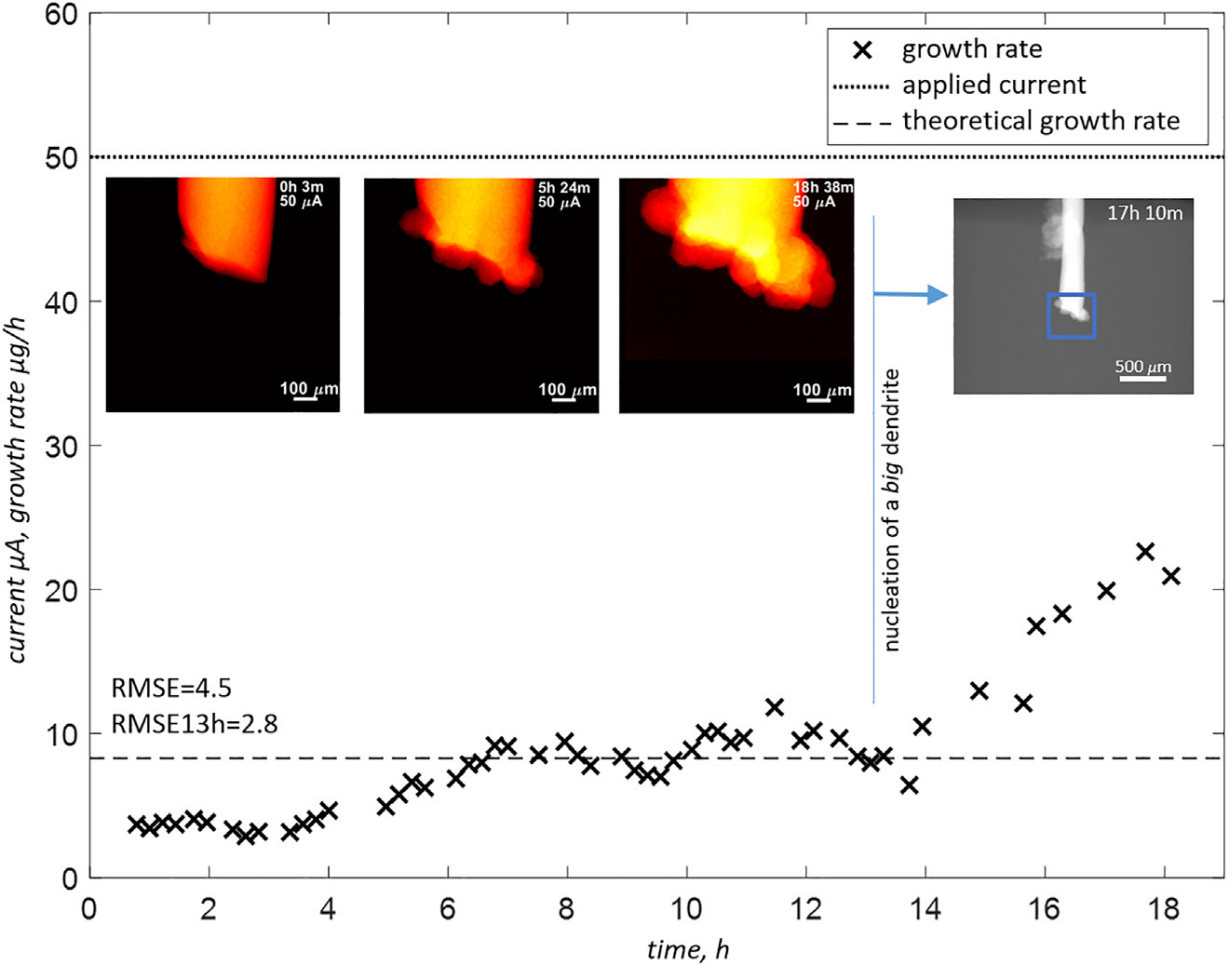
Material deposition visualization for sample zw_07(NaCl) Overview radiogram is shown to demonstrate the big dendrite which appeared at the end of the experiment. Note that two RMSE values are provided – one for the complete experiment and second (RMSE13h) excluding the time period with elevated growth rates.

It is also visible for sample *zw_07*, operating within the near-neutral environment as shown in Figure 6. For most of the time, the material exhibits smooth, non-dendritic plating and shows good agreement with theoretical values (RMSE13h in Figure 6). At a certain point in time, however, the growth rate is suddenly increased. The reason for this is revealed by the overview radiogram showing the nucleation of a very large dendrite, which penetrated even inside the capillary and completely changed the dynamics of the system. It is not clear why the material suddenly started to deposit in a completely new location. Nevertheless, it is a good example demonstrating the importance of multi-scale analysis, showing that focusing only on a highly magnified area might lead to confusion or even wrong conclusions if not assisted by (at least) qualitative assessment from at larger field of view.

**Figure 7:**
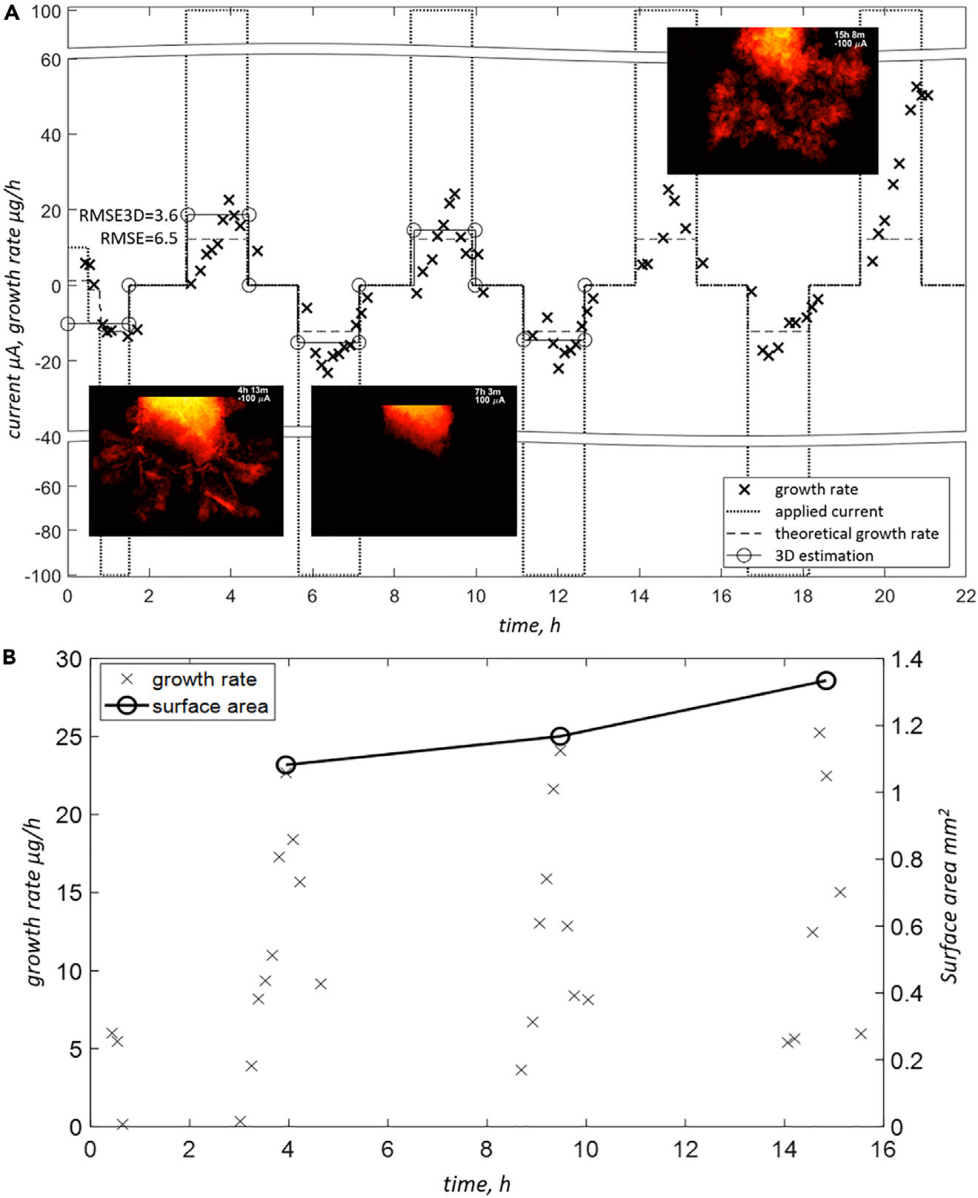
Material deposition visualization for sample zw_09(KOH) Electrode evolution for sample zw_09(KOH) (A) and surface area changes for the first 3 sequences of stripping-plating (B). Note that 3D acquisitions are not available for the last 1.5 cycle. Single RMSE value was calculated for all cycles including positive and negative currents. Last half-cycle was considered the outlier and excluded from RMSE calculations. RMSE3D represents the difference between growth rates obtained from the 3D measurement and theoretical values.

Sample *zw_09*, operating within KOH, was measured under cycling conditions and with longer OCV times to enable additional 3D acquisitions. The applied current was alternating between 100 and -100 μA. Interestingly, as in Figure 7A, we see behavior similar to one observed at lower currents for samples *zw_01* and *zw_07* operated in near-neutral electrolyte. Plating/stripping rate increases slowly and then oscillates around the plateau. This time, however, the effect is more pronounced, and with every cycle, the peaks become higher. This might be attributed to the development of a complex morphology characterized by a larger active surface area. In Figure 7B, the monotonic increase of surface area, obtained from tomograms acquired at the end of each stripping-plating sequence, confirms this speculation. At the end of the experiment, again – in the last cycle, the system became less stable, and strongly increased growth rates were observed. Sparsely (in time) recorded tomograms provided growth rates (“3D estimation” in Figure 7A) that are in good agreement with theoretical values. Note, however, that radiograms contain the same information, which is averaged over much shorter period and hence providing some insight into the dynamics of the plating/stripping process.

It was enough to just shift the electrolyte into a mild acidic range (ZnSO_4_) while keeping the same experimental protocol, to observe a completely different behavior of the sample. The strictly dendritic growth of *zw_09*(KOH) changed to smooth deposition in *zw_10*(ZnSO_4_), without a single dendrite reaching more than a few microns outside of the initial envelope. It is interesting to observe, especially at 100 μA - high current, which, in case of near-neutral and alkaline electrolytes, was always triggering rapid, random growth of dendrites in every possible direction.

## Discussion

Note that the quantification performed in this work serves only to demonstrate the capabilities of the method, and any electrochemical conclusions must be taken with a grain of salt. To learn about the mechanism behind the Zn dendrite formation under different conditions, the reader is encouraged to explore numerous existing studies on this topic.^8^^,^^12^^,^^45^^,^^46^^,^^47^^,^^48^^,^^49^^,^^50^ Nonetheless, with a little leverage from the current state of art, some conjectures might already be formed.

**Figure 8:**
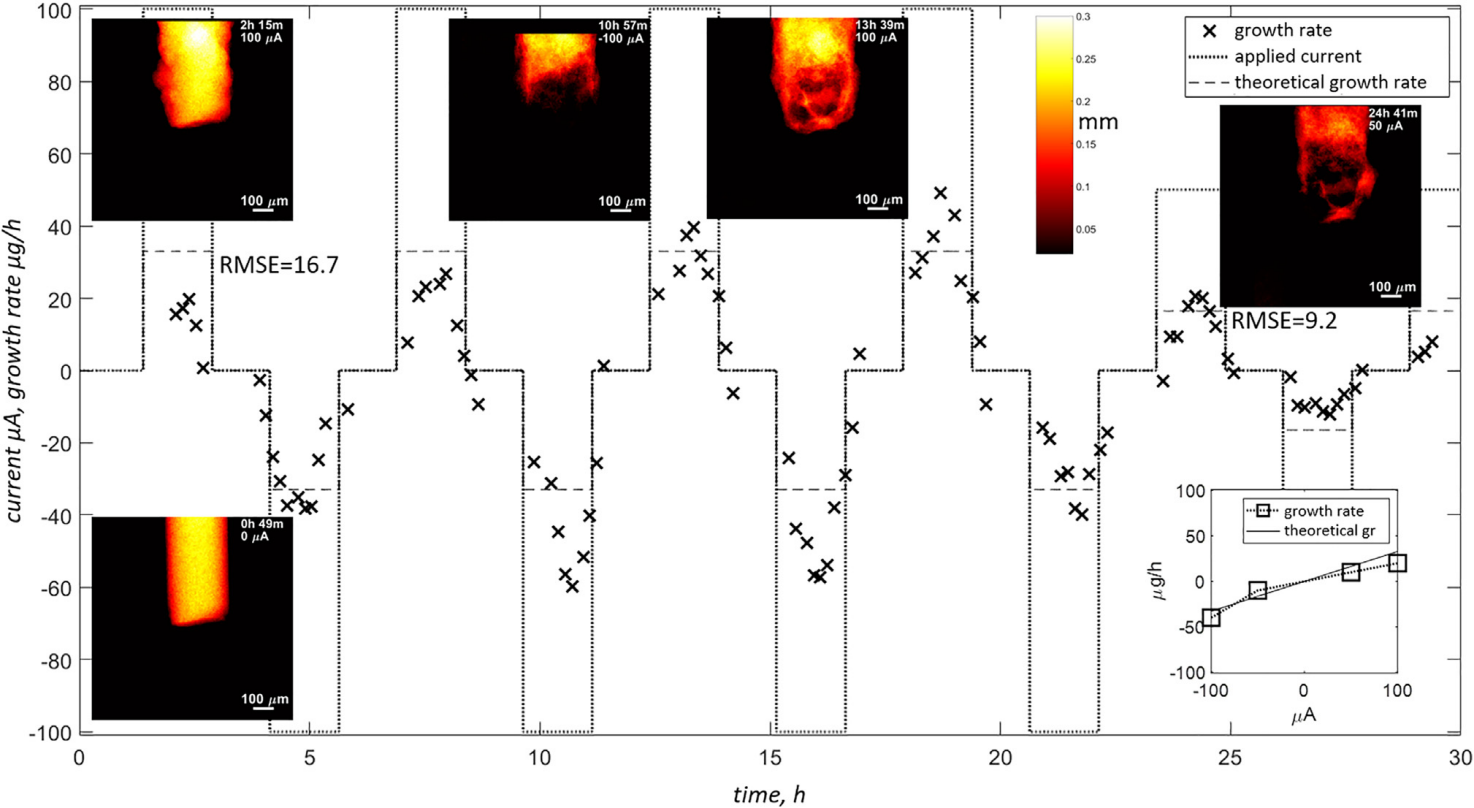
Electrode evolution for sample zw_10(ZnSO4) Note that the graph at the bottom right corner was created by averaging the measured growth rates for each current. Each RMSE value represents all cycles performed at the same absolute current.

The shape change and dendritic growth of Zn deposition are generally triggered by the following factors: inhomogeneous current distributions within the electrode, local reaction zones, non-uniform dissolution and deposition of Zn, and concentration gradients at the electrode surface.^45^^,^^46^^,^^47^^,^^48^ Note that the qualitative assessment of the evolving electrodes, as shown in Figures 5, 6, 7 and 8, already confirms the influence of the local morphology on system’s dynamics. With time, especially in the case of the dendritic type of growth, more agglomerations that form complex topology appear and act as nucleation sites and growth exhibitors. This results in a higher deviation from the predicted growth rate and is pronounced especially for *zw_09*(KOH), as shown in Figure 7, after 20 h of the experiment.

There are several strategies to mitigate the dendrite growth in Zn electrodes among which, modification of the Zn deposition by employing organic and inorganic additives for Zn electrode and electrolyte is the most common method especially in alkaline and acidic solutions.^51^^,^^52^Neutral or near-neutral electrolytes, on the other hand, provide a promising alternative with reduced dendrite formation due to the significantly lowered solubility of Zn.^53^^,^^54^ In such electrolytes, additives are also employed either to improve the Zn deposition behavior even more or to enhance the activity of Zn.^55^^,^^56^ In this study, however, *zw_07* and *zw_01*, which were immersed in NaCl with the addition of glycine (near-neutral), still showed a tendency toward dendritic growth unless the applied current was very low (25 or 10 μA). This could be due to the insufficient concentration of the additive or its neutralization by the initial addition of the Zn into the electrolyte. Moreover, the low statistical power of only a few samples should not be overlooked when drawing strong conclusions. The authors plan to perform more experiments to gain sufficient statistics for a clear insight into the process and finally explain how different additives modify the morphological dynamics of each system, i.e., alkaline, near-neutral, and acidic.

**Figure 9:**
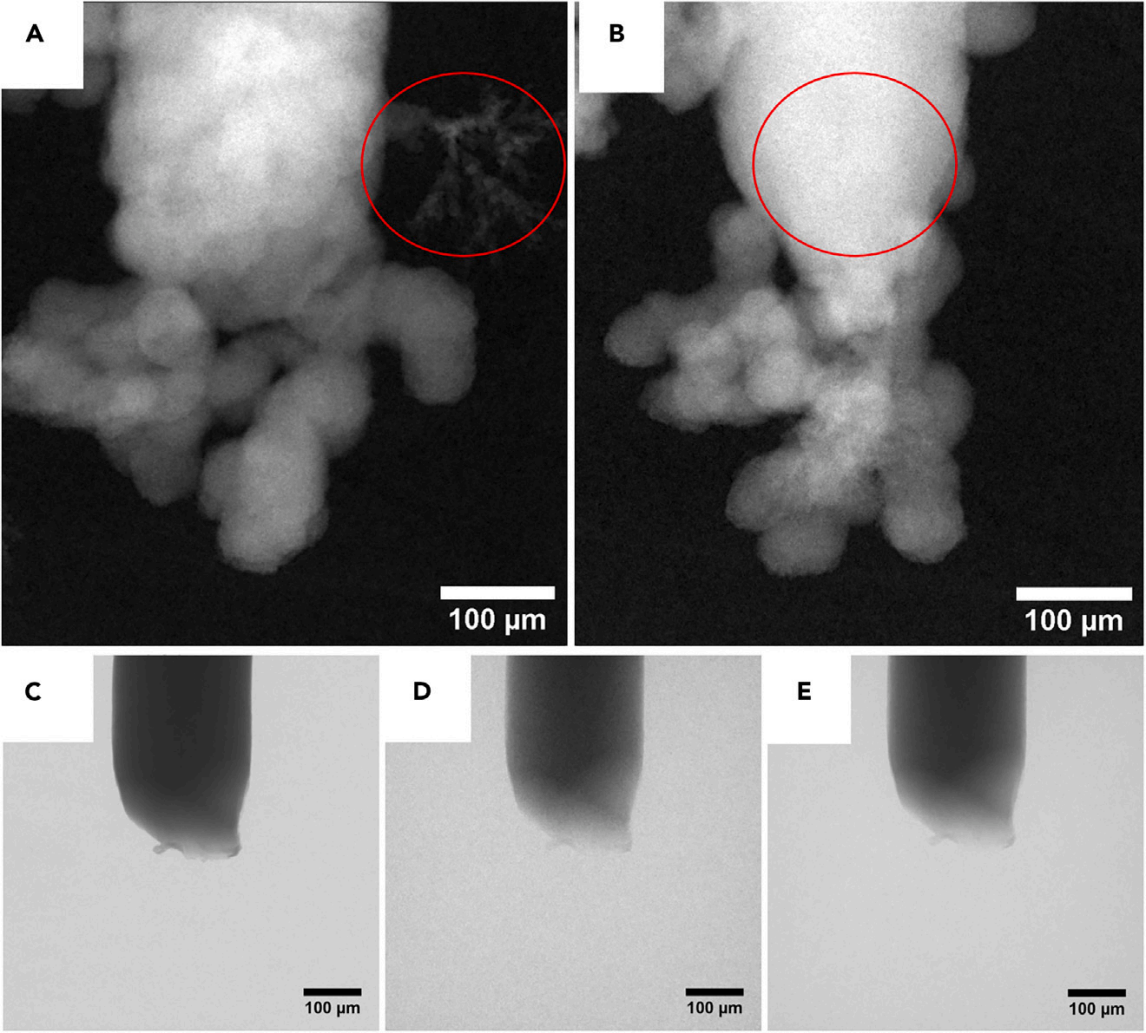
Challenges introduced by the liquid alectrolyte and the three-dimensional nature of the dendrites Two orthogonal radiograms taken at 90° (A) and 0° (B) with feature visible only on one, although contributing to total attenuation in both cases. Degradation of the image quality due to liquid is more apparent when comparing raw radiogram of the pristine cell without liquid inside (C) to the one filled with electrolyte (D). At reduced energy (from source voltage 100kV-60kV) and longer exposure (from 8 to 60 s) only SNR improves but interfaces are still blurred (E).

The presented measurement protocol is meant to assess existing concepts using qualitative, visual inspection of the process, as well as quantitative descriptors such us deposition/dissolution rate of material. Thanks to the two-dimensional nature of the measurement, the achieved temporal resolution is sufficient to study electrode evolution even at high currents. Additionally, 3D acquisitions, although distributed very sparsely along the timeline, can help interpret the 2D data. Note, however, that radiograms are captured at two orthogonal orientations, which is helpful if, for instance, deposition is highly anisotropic. Such an example is shown in Figures 9A and 9B, where dendrites are expanding in the direction of scan integrals at 0°. Although the amount of the material deposited can still be estimated, the actual topology is lost since the distribution of the material along scan line integrals cannot be retrieved. The acquisition of two orthogonal radiograms only costs a few additional seconds, but, in return, reveals the hidden features and reduces the risk of incorrect interpretation. Note that Figures 9C-9E visualizes the problem already mentioned at the beginning of this section, where scattering from liquid, together with growing structures, caused additional degradation in image quality. Wire in the Figures 9C-9E was cut and damaged at the tip to generate additional features. It was then scanned without (Figure 9C) and with electrolyte inside (Figure 9D). The raw radiograms (white pixels representing high transmission) show that not only does the SNR drop, but additional blurring occurs at the interfaces as well, and it cannot be mitigated by applying lower energies and longer exposures (Figure 9E).

### Conclusions

A robust measurement protocol was developed, employing laboratory XCT to study the evolution of electrode materials in operation. The combination of two- and three-dimensional imaging revealed changes in morphology with sufficient temporal and spatial resolution and enabled quantification with respect to growth rate and surface area. The protocol was successfully demonstrated within three different environments and at several growth rates defined by applied currents. Although the authors do not intend to provide any electrochemical interpretation of the observed electrode’s evolution, it must be highlighted that the calculated deposition and dissolution rates are in good agreement with theoretical values. This indicates that the approach might be applicable for evaluating existing models, especially related to Zn, which was chosen in this work as one of the promising candidates to become an important medium for primary and secondary energy storage.

### Limitations of the study

Obviously, laboratory devices can’t compete with synchrotron radiation facilities. Therefore, depending on the material being studied, this protocol requires from the user to find a proper balance between sufficient SNR and temporal resolution. Additionally, each 3D scan requires the experiment to be put on hold (OCV) for at least 1 h, or more, depending on the density of the material being examined. Moreover, the results are dependent on the quality of image registration, and in case of lacking features, for example, when dealing with large, homogeneous regions, it might lead to a strong under/over-estimation of deposition rate. The amount of data collected also plays a crucial role, as demonstrated in Table 2, where estimated, local thickness was compared to results from 3D acquisition.

The limited flux of laboratory XCT scanners, will always impose additional restrictions, such as the maximum applied current. As seen in Figure 5, at 100 μA, the standard deviation of the calculated growth rate became significant. To some extent, this can be improved by better cell design allowing more efficient gas release and therefore preventing bubbles from staying within the field of view. Nevertheless, speeding up the process by increasing the current will always lead to the standard dilemma of the XCT scanner operator, forcing to choose between low SNR and pronounced motion artifacts. In the future, this can be addressed by compressed sensing^57^ and super resolution^58^^,^^59^ techniques. However, to achieve this, changes must be made to the protocol itself, and sufficient ground-truth data must be acquired first to train the model for each type of sample. Nevertheless, one should always consider the availability of XCT scanners, lack of time restrictions, and low operational costs. Therefore, authors strongly believe that custom acquisition protocols, like the one presented here, can significantly improve usability of the laboratory devices.

## STAR★Methods

### Key resources table

**Table:** 

REAGENT or RESOURCE	SOURCE	IDENTIFIER
**Software and algorithms**		

MATLAB R2022b	MathWorks USA	https://es.mathworks.com/products/matlab.html
Scripts repository	Forschungszentrum Julich	https://jugit.fz-juelich.de/iek-9-functional-materials/sharedendrites
Python 3.11	Python Software Foundation	www.python.org

**Chemicals, Peptides, and Recombinant Proteins**		

NaCl crystals	Merc-Milipore	https://www.merckmillipore.com/DE/de/product/Sodium-chloride, MDA_CHEM-106404
KOH pellets	Fluka	https://www.fishersci.de/shop/products/potassium-hydroxide-acs-reagent-honeywell-7/15672140
ZnO	Sigma-Aldrich	https://www.sigmaaldrich.com/DE/de/product/sigald/96479
Glycine	Alfa Aesar	https://www.alfa.com/en/catalog/A13816/

**Other**		

Zinc wire 0.25 mm	Alfa Aesar	https://www.alfa.com/en/catalog/012055/

### Resource availability

#### Lead contact

Further requests for resources should be directed to the lead contact, Krzysztof Dzieciol (k.dzieciol@fz-juelich.de).

#### Materials availability

This study did not generate new unique reagents.

#### Method details

Since this work is purely methodological, for details regarding the experimental setup and evaluation steps, the reader is referred to the main text.

## Data Availability

The reader is encouraged to download the source code, measurement protocol and CAD model of the designed cell linked in the key resources table. Upon request, authors are happy to share all the acquired imaging data.
